# Salivary changes and dental caries as potential oral markers of autoimmune salivary gland dysfunction in primary Sjögren's syndrome

**DOI:** 10.1186/1472-6890-5-4

**Published:** 2005-03-01

**Authors:** Anne Marie Lynge Pedersen, Allan Bardow, Birgitte Nauntofte

**Affiliations:** 1 Department of Oral Medicine, Clinical Oral Physiology, Oral Pathology and Anatomy, and Copenhagen Gerodontological Research Centre, Faculty of Health Sciences, University of Copenhagen, Norre Allé 20, 2200 Copenhagen N, Denmark

## Abstract

**Background:**

the classification criteria for primary Sjögren's syndrome (pSS) include a number of oral components. In this study we evaluated if salivary flow and composition as well as dental caries are oral markers of disease severity in pSS.

**Methods:**

in 20 patients fulfilling the American-European Consensus criteria for pSS and 20 age-matched healthy controls whole and parotid saliva flow rates and composition, measures of oral dryness, scores of decayed, missing and filled tooth surfaces (DMFS), periodontal indices, oral hygiene, and dietary habits were examined.

**Results:**

in pSS, salivary flow rates, pH, and buffer capacities were lower, and DMFS, salivary sodium and chloride concentrations higher than in the healthy controls. DMFS also correlated inversely to salivary flow rates and positively to oral dryness. Apart from slightly increased gingival index, and more frequent dental visits in pSS, the periodontal condition, oral hygiene or sugar intake did not differ between these two groups. In pSS, findings were correlated to labial salivary gland focus score (FS) and presence of serum-autoantibodies to SSA/SSB (AB). The patients having both presence of AB and the highest FS (>2) also had the highest salivary sodium and chloride concentrations, the lowest salivary phosphate concentrations, lowest salivary flow rates, and highest DMFS compared to those with normal salivary concentrations of sodium and chloride at a given flow rate.

**Conclusion:**

the salivary changes observed in some pSS patients reflect impaired ductal salt reabsorption, but unaffected acinar transport mechanisms, despite low salivary secretion. Our results suggest that changes in salivary flow and composition as well as dental caries may serve as potential markers of the extent of autoimmune-mediated salivary gland dysfunction in pSS. The study also indicates that the ductal epithelium is functionally affected in some pSS patients, which calls for future pathophysiological studies on the mechanisms underlying this impaired salt reabsorption.

## Background

Primary Sjögren's syndrome (pSS) is a common systemic autoimmune disease that mainly affects middle-aged women. The exact aetiology remains elusive, and appears multifactorial. The disease is characterised by a chronic periductal lymphocytic infiltration of the exocrine glands, predominantly of the lacrimal and salivary glands. The chronic inflammation is assumed to lead to glandular tissue destruction, resulting in keratoconjunctivitis sicca and hyposalivation and associated symptoms of ocular and oral dryness [1,2].

The salivary dysfunction in pSS is of significant clinical importance and may cause chronic oral discomfort as well as compromised oropharyngeal functions [3-5]. Subsequently, the patients' well-being and oral health-related quality of life are also impaired [5,6]. Cross-sectional studies have shown that patients with pSS have a high caries experience with lesions usually located on the cervical surfaces of the teeth, as well as early dental loss due to caries [3,7-10], despite good oral hygiene [11]. Dental caries, which is a local, but also a multifactorial disease, arises from a concerted action of the aetiological factor (dental plaque) and determinants including salivary factors, medication, systemic disease as well as behavioural factors [12]. However, the selective significance of salivary factors on dental caries in pSS remains an open question. In light of the impaired saliva formation in pSS it is noteworthy that an exact cut-off value for salivary flow and/or separate salivary constituents predicting the risk of developing dental caries has not yet been agreed on. In the diagnosis of Sjögren's syndrome (SS), an unstimulated whole saliva flow rate of ≤ 1.5 ml/15 min is currently considered pathological [13], but at this point the caries process may have been going on for years revealed by the presence of a high number of decayed, missed and filled teeth [3,7-10].

The American-European Consensus Classification Criteria for pSS include evaluation of salivary gland dysfunction by means of parotid sialography, salivary gland scintigraphy, whole saliva sialometry and labial salivary gland biopsy as well as questionnaire concerning symptoms of oral dryness [13]. The purpose of this study was to evaluate parotid flow rate, whole and parotid saliva composition and dental caries as potential oral markers of disease severity in patients with pSS. Thus we hypothesised that patients with pSS exhibit specific changes in salivary flow and composition due to presence of focal lymphocytic infiltration in their labial salivary glands (FS) and/or serum autoantibodies to Ro (SSA) and/or La (SSB) antigens (AB) as compared to age- and gender-matched healthy controls with normal salivary gland function. We further hypothesised that in patients with pSS dental caries is related to impaired salivary flow and associated changes in saliva composition.

## Methods

The study included 20 randomly selected patients with pSS, who attended the Department of Oral Medicine, University of Copenhagen, for routine follow-up examinations. They were diagnosed according to the Copenhagen criteria, but also met the American-European Consensus Classification Criteria for pSS [13,14]. All patients had complaints of dry eyes and dry mouth according to the questionnaire of the American-European classification criteria [13] as well as evidence of keratoconjunctivitis sicca. Eighteen patients had unstimulated whole saliva flow rate (UWS) ≤ 1.5 ml/15 min. Two patients had UWS >1.5 ml/15 min, but otherwise fulfilled the criteria. Fourteen patients had a positive labial salivary gland biopsy with focus score ≥ 1 as well as presence of AB. Among the remaining 6 patients, 2 had FS, but not presence of AB, and 4 patients had presence of AB, but not FS. The labial salivary glands of the latter 4 patients displayed chronic sialoadenitis where inflammatory cells were observed but did not form any foci. In all 20 patients, estimation of the presence of serum autoantibodies was performed within the same timeframe as the saliva collections and the clinical assessments. In 16 of these patients labial salivary gland biopsies were performed at the same time. The remaining 4 patients refused a labial salivary gland rebiopsy, as they had their diagnosis of pSS for 25, 23, 16 and 10 years, respectively.

In order to understand the underlying salivary pathophysiology in pSS, the study also included 20 healthy controls selected among persons attending the Institute of Odontology for dental treatment to obtain reference values of normalcy. The selection was based on the requirements that controls had no present or past medical history of systemic disease, did not take any medication including contraceptives and hormonal replacement, had no current use of tobacco products, and matched the pSS patients with regard to age.

### Clinical examination

One examiner (AMLP) conducted the whole saliva collections, interviews, and oral clinical examinations. About 2 hours after collection of whole saliva, another examiner (AB) conducted collections of parotid saliva. In order to minimize the influence of circadian cycle on salivary secretion and composition all procedures were carried out in the same order and at a fixed time of the day, i.e. between 9.00 and 11.45 a.m. The local Danish Ethical Committees approved the study protocol, and all participants completed an informed consent form according to the Declaration of Helsinki. The number of decayed, missing and filled surfaces (DMFS) was recorded excluding third molars as previously described in details [5]. Plaque and gingival indices and periodontal probing pocket depth were determined at four sites per tooth on six index teeth (16, 21, 24, 36, 41, and 44) [8]. Each subject underwent a standardised interview including inquiries on medical disease symptoms, dental history, dental visits, medication, tobacco smoking, dietary (especially regarding daily sugar intake) and oral hygiene habits. Furthermore, symptoms of oral dryness were assessed by means of a categorised questionnaire (based on Beck's inventory scale, item 9 [15]) with four degrees of severity (scores 0–3), as previously described [8]).

### Collection of whole and parotid saliva

Unstimulated whole saliva (UWS) and paraffin-stimulated whole saliva (SWS) were sampled over a 15-min and 5-min period, respectively, as described previously [8]. Unstimulated parotid saliva (UPS) and stimulated parotid saliva (SPS) were sampled over an average period of 20 min and 5 min, respectively. A saliva collection set-up impermeable to CO_2 _was used [16]. Stimulation of the parotid saliva was initiated by applying 1 ml of 1% citric acid to the dorsal part of the tongue every 15 sec. The flow rate, given with 2 decimal places, was determined by weighing the saliva-collecting cup, tube, and syringe before and after saliva collection, and expressed as ml/min [8]. The subjects were instructed to refrain from eating, drinking, smoking, and any oral hygiene for 2 h preceding the saliva sampling.

### Sialochemical analysis

Concentrations (mM) of sodium, potassium and total calcium were determined by atomic absorption spectroscopy [16,17]. Concentrations (mM) of chloride were measured by coulometric titration and total phosphate (mM) by the molybdic reaction [16,17]. Total protein (μg/ml) was measured by the Coomassie reaction. Amylase activity (stated as the catalytic activity of the enzyme, i.e., kat = mole/s and given in μkat/l, where one μkat/l corresponds to 60 U/l) was measured by means of the Phadebas™ test kit. Saliva was diluted 1000 times more than usually recommended for plasma by the test kit. The volume of saliva required for complete sialochemical analysis was 240 μl corresponding to an average collection period for UPS of less than 20 min and for SPS less than 5 min for the pSS patients.

Parotid saliva pH, P_CO2_, buffer capacity, and saturation with regard to hydroxyapatite Parotid saliva pH and P_CO2 _were determined on an ABL 605 blood gas analyser (Radiometer™), and the HCO_3_^- ^concentration was calculated from the pH and Pco_2 _values as previously described [17]. The parotid saliva buffer capacity (β), which originated from the HCO_3_^- ^and phosphate buffer systems, was calculated individually for each saliva sample at the pH value of the sample [18]. Briefly, the buffer capacity for each buffer system was calculated as:

2.3 [C] * ([C_B_]/[C]) * (1-([C_B_]/[C]))

Where ([C]) states the total concentration of the buffer system and ([C_B_]) states the concentration of the base in the buffer system. The concentrations of the bases was calculated as previously described [16] and the pK values used for the bicarbonate and phosphate buffer systems was 6.1 for carbonic acid [16,17] and 6.8 for H_2_PO_4_^- ^[19]. The sums of calculated β from both the HCO_3_^- ^and phosphate buffer systems are in this study denoted as saliva β. At pH values above 5 (counting for all except one sample) the contribution from the protein buffer system, relative to the HCO_3_^- ^and phosphate buffer systems, was considered negligible [16] and therefore not included in the saliva β.

The degree of saturation (DS_HAP_) of saliva with respect to hydroxyapatite, i.e. the calcium phosphate salt and main mineral of the tooth tissues, was calculated according to the method described by Schmidt-Nielsen [19]. The negative logarithm of the ionic product of hydroxyapatite (pI_HAP_) was calculated from the saliva p [Ca^2+^], p [HPO_4_^2-^], and p [H^+^]. The negative logarithm of the solubility product of hydroxyapatite (pSP_HAP_) was calculated by the constant given by Schmidt-Nielsen [19] and the ionic strength of each saliva sample. Given the relatively low ionic strength of the saliva samples analysed (0.039 ± 0.020) we found this simple method for calculation of DS_HAP _acceptable. Finally, the critical pH of parotid saliva, i.e. the pH value at which saliva is saturated (i.e., neither super- nor undersaturated) with respect to hydroxyapatite (pI_HAP _equal to pSP_HAP_) was calculated.

### Statistical analysis

Differences in the salivary and clinical parameters between the patient and control groups were analysed by Wilcoxon rank-sum test (categorised variables) and two-sampled *t*-test (numerical variables). Fisher's test (less than ten in one category) and the chi-squared test (more than ten) were used for analysis of distributions between the two groups. Associations between variables were analysed by the Spearman rank order correlation analysis (*r*_s_). In order to find the best predictor for a given outcome, multiple regression analysis was used with stepwise backward elimination with the adjusted R-squared values given. Significance was selected at a level of *P *≤ 0.05.

## Results

### Clinical findings

Table 1 summarises some of the anamnestic data and results of self-assessed oral dryness of the 20 female patients with pSS and 20 age-matched healthy females included in this study. Sixteen pSS patients were taking prescribed medicines on a regularly daily basis. Seven of these patients were taking medicines known to impair saliva secretion and/or cause compositional changes of saliva [20]. These included antihistamines and antidepressants that cause reduction in salivary flow due to inhibition of the muscarinic cholinergic receptors [21], and blockers of β-adrenoceptors that lead to impaired protein secretion [22]. However, none of these medicines are known to cause specific changes in the inorganic salivary composition. The 7 patients taking "xerogenic" medicines all had symptoms of oral dryness and/or were diagnosed as pSS with hyposalivation (n = 5) before their current medication was commenced. We therefore found no significant association between the intake of medicines, including the number of medicines, the salivary flow rates, focus scores, and serum autoantibodies. All the pSS patients complained of dry mouth. In pSS, a significant inverse correlation was found between scores of oral dryness and the SWS (*r*_*s *_= -0.50, *P *= 0.025), but only a tendency toward a relationship between the oral dryness and UWS, UPS and SPS. On the other hand, in both groups as a whole, displaying large variations in oral dryness and salivary flow rates, the subjective measures were significantly inversely correlated to UWS (*r*_*s *_= -0.83, *P *< 0.001), SWS (*r*_*s *_= -0.81, *P *< 0.001), UPS (*r*_*s *_= -0.74, *P *< 0.001), and SPS (*r*_*s *_= -0.77, *P *< 0.001).

**Table 1:** Anamnestic data and results of self-assessed oral dryness of the female patients with primary Sjögren's syndrome (pSS) and the female healthy controls. Results are given as number of patients or scores (yes/no) and as means ± SD.

	pSS (*n *= 20)	Healthy controls (*n *= 20)	P-value
Age (years)	60 ± 15	56 ± 13	0.437^a^
Duration of disease (years)*	6 ± 7	0	NA
Duration of symptoms (years)	10 ± 7	0	NA
Xerogenic medicines (yes/no)^†^	7/13	0/20	NA
Smokers (yes/no)^§^	5/15	0/20	NA
Cigarettes per day (smokers only)^§^	7 (2–20)	0	NA
Tooth brushing (times per day)	3 ± 1	2 ± 1	0.001^a^
Dental floss and/or toothpicks daily (yes/no)	16/4	15/5	1.000^b^
Dental visits per year (number)	3 ± 1	2 ± 1	0.001^a^
Oral dryness (score 0/1/2/3)	0/3/4/13	20/0/0/0	<0.001^b^

* The time from established diagnosis to present examination. ^†^Patients were interviewed regarding their intake of medicines. Their intake of xerogenic medicines included antidepressants, antihistamines and β-blocking agents [20]. ^§^Patients were also interviewed regarding their smoking habits, since cigarette smoking has been shown to reduce the extent of labial salivary gland lymphocytic infiltration [23] and to have a negative impact on periodontal status. Smoking, however, has no impact on salivary flow or composition [24].

P-values obtained by a two-sample t-test (a) and Fisher exact test (b). NA; not analyzed.

The results of the clinical examination are shown in Table 2. In spite of the fact, that the patients brushed their teeth with fluoride-containing toothpaste and visited their dentist more frequently than the healthy individuals (Table 1), they had a significantly higher number of decayed, missed and filled teeth and a higher gingival index. No significant differences were found between the two groups in terms of plaque index (frequency distribution of scores), the periodontal probing pocket depth or the regular daily use of dental floss and/or toothpicks. Apart from avoiding acidic, spicy, crunchy and dry foods the patients' dietary habits (including sugar intake) did not differ from those of the healthy controls. Neither the time since pSS diagnosis, nor the duration of disease symptoms or the age, were correlated to the flow rates. Moreover, plaque- and gingival indices as well as periodontal probing pocket depth were not correlated to the salivary flow rates, time since pSS diagnosis, duration of disease symptoms, DMFS, or age. In the groups as a whole, however, a positive association between the gingival index and age was found (*r*_*s *_= 0.33, *P *= 0.04).

**Table 2:** Oral findings in the patients with primary Sjögren's syndrome (pSS) and the healthy controls. Results are given as number of patients or scores (yes/no) and in medians (ranges).

	pSS (*n *= 20)	Healthy controls (*n *= 20)	P-value
No. of teeth	22 (6–28)	28 (0–28)	0.011^a^
DMFS*	83.0 (25–140)	43.0 (10–140)	0.001^a^
Distribution of D/M/FS	23/745/1020	14/305/662	<0.001^b^
Subjects with dentures (yes/no)	4/16	7/13	0.480^c^
Distribution of PI (no. of 0/1/2/3 scores)	245/179/54/2	244/152/60/0	0.272^b^
Distribution of GI (no. of 0/1/2/3 scores)	346/116/16/2	330/95/31/0	0.034^b^
Probing pocket depth (PPD, mm)	2 (1–4)	2 (1–3)	0.523^a^

*D/M/FS, decayed, missed, filled surfaces; PI, plaque index; GI, gingival index. P-values obtained by Wilcoxon rank sum test (a), Chi^2^- test (b), and Fisher exact test (c).

### Salivary flow and composition

As shown in Tables 3 and 4, the pSS patients had significantly lower UWS, SWS, UPS and SPS than the healthy controls. All pSS patients and one single healthy individual (without symptoms or clinical signs of local or systemic disease) had with UWS ≤ 0.10 ml/min and/or SWS ≤ 0.70 ml/min, which are the cut-off values for hyposalivation [13,25]. In 8, 11 and 5 patients, UWS, UPS and SPS, respectively, were close to or equal to 0 ml/min. In both the patient and control group UWS and SWS were mutually correlated (*r*_*s *_= 0.79, *P *< 0.001 and *r*_*s *_= 0.45, *P *< 0.05, respectively), but only in pSS a mutual correlation was obtained between UPS and SPS (*r*_*s *_= 0.74, *P *< 0.001).

**Table 3:** Unstimulated (UWS) and stimulated (SWS) whole salivary flow rate and composition in the patients with primary Sjögren's syndrome (pSS, *n *= 20) and the healthy controls (*n *= 20). Results are given in median (range).

	UWS			SWS		
	pSS	Healthy controls	P-value	pSS	Healthy controls	P-value

Flow rate (ml/min)	0.02 (0.00–0.23) (*n *= 20)	0.39 (0.06–1.10) (*n *= 20)	<0.001	0.14 (0.01–1.66) (*n *= 20)	1.40 (0.54–2.82) (*n *= 20)	<0.001
Na^+ ^(mM)	12.0 (8.0–47.0) (*n *= 11)	8.0 (4.5–17.0) (*n *= 20)	0.008	16.0 (8.0–59.0) (*n *= 12)	10.3 (5.5–30.0) (*n *= 20)	0.020
K^+ ^(mM)	22.2 (5.1–48.0) (*n *= 11)	21.9 (6.8–33.4) (*n *= 20)	0.536	21.6 (15.2–34.6) (*n *= 12)	22.0 (11.5–26.5) (*n *= 20)	0.533
Total calcium (mM)	2.0 (1.3–3.2) (*n *= 7)	1.7 (0.5–2.8) (*n *= 19)	0.285	1.3 (0.7–1.9) (*n *= 12)	1.3 (0.8–2.5) (*n *= 20)	0.558
Cl^- ^(mM)	25.8 (16.0–62.0) (*n *= 10)	18.3 (5.2–26.0) *(n *= 19)	0.003	22.6 (13.5–58.6) (*n *= 11)	16.1 (10.5–28.6) (*n *= 20)	0.037
Total phosphate (mM)	6.0 (1.2–15.0) (*n *= 9)	6.8 (2.4–11.7) (*n *= 19)	0.961	4.4 (2.4–8.5) (*n *= 11)	4.1 (1.6–11.5) (*n *= 20)	0.606
Total protein (mg/ml)	3.00 (1.52–8.84) (*n *= 8)	3.29 (1.36–6.25) (n = 19)	0.560	3.50 (0.421–8.93) (*n *= 12)	3.04 (1.23–5.54) (*n *= 20)	0.491
Total protein output (mg/min)	0.210 (0.06–0.80) (*n *= 8)	1.09 (0.50–2.38) (*n *= 19)	<0.001	0.95 (0.11–4.33) (n = 12)	3.88 (2.35–8.06) (*n *= 20)	<0.001
Amylase activity (μkat/l)	365 (0–4500) (*n *= 10)	980 (0–8940) (*n *= 19)	0.155	1490 (0–4080) (*n *= 12)	2280 (0–5890) (*n *= 20)	0.199

P-value obtained by Wilcoxon's rank sum test.

**Table 4:** Unstimulated (UPS) and stimulated (SPS) parotid salivary flow rates, composition including pH, P_CO2_, buffer capacity, degree of saturation with regard to hydroxyapatite, and critical pH in the patients with primary Sjögren's syndrome (pSS, (*n *= 20)) and the healthy controls (*n *= 20). Results are given in medians (range).

	UPS			SPS		
	pSS	Healthy controls	P-value	pSS	Healthy controls	P-value

Flow rate (ml/min/gland)	0.00* (0.00–0.04) (*n *= 20)	0.04 (0.00–0.13) (*n *= 20)	<0.001	0.10 (0.00–0.87) (*n *= 20)	0.72 (0.17–1.57) (*n *= 20)	<0.001
pH	5.5 (4.9–6.2) (*n *= 7)	6.1 (5.4–6.6) (*n *= 14)	0.008	6.8 (5.3–7.6) (*n *= 13)	7.1 (6.8–8.0) (*n *= 19)	0.020
P_CO2 _(KPa)	2.5 (2.1–4.8) (*n *= 7)	3.3 (0.5–7.9) (*n *= 14)	0.455	3.5 (1.4–7.5) (*n *= 13)	5.5 (0.3–10.0) (*n *= 19)	0.021
						
Na^+ ^(mM)	7.0 (0.0–111.0) (*n *= 9)	0.0 (0.0–8.5) (*n *= 19)	0.002	15.5 (3.5–93.0) (*n *= 15)	7.5 (0.0–65.0) (*n *= 20)	0.034
K^+ ^(mM)	24.5 (4.5–52.5) (*n *= 9)	29.0 (17.5–62.0) (*n *= 19)	0.209	26.5 (12.0–53.0) (*n *= 15)	23.0 (18.0–40.0) (*n *= 20)	0.689
Total calcium (mM)	1.0 (0.4–2.4) (*n *= 9)	1.0 (0.6–4.1) (*n *= 16)	0.887	1.1 (0.6–1.8) (*n *= 13)	0.8 (0.4–1.4) (*n *= 20)	0.008
						
Cl^- ^(mM)	22.5 (5.0–100.5) (*n *= 9)	19.0 (12.0–43.0) (*n *= 19)	0.588	27.0 (12.5–94.0) (*n *= 14)	15.5 (9.0–59.0) (*n *= 20)	0.037
Bicarbonate (mM)	0.2 (0.0–0.8) (*n *= 8)	0.8 (0.1–3.5) (*n *= 14)	0.017	3.6 (0.1–26.8) (*n *= 12)	11.3 (3.8–43.9) (*n *= 19)	0.025
Total phosphate (mM)	5.6 (2.3–15.0) (*n *= 7)	9.3 (4.1–18.9) (*n *= 16)	0.066	6.1 (2.1–8.2) (*n *= 11)	5.2 (3.2–16.9) (*n *= 20)	0.577
						
Total protein (mg/ml)	1.11 (0.47–8.97) (*n *= 9)	2.02 (0.64–5.34) (*n *= 18)	0.316	1.33 (0.62–6.66) (*n *= 14)	1.60 (0.84–4.19) (*n *= 20)	0.589
Total protein output (mg/min)	0 (0–0.18) (*n *= 9)	0.07 (0–0.30) (*n *= 18)	<0.001	0.13 (0–1.38) (*n *= 14)	1.30 (0.29–5.42) (*n *= 20)	<0.001
Amylase activity (μkat/l)	7760 (3700–21200) (*n *= 7)	5815 (1670–27140) (*n *= 18)	0.193	5220 (560–17240) (*n *= 13)	5520 (710–14710) (*n *= 20)	0.624
						
β(mmol H^+^/l^. ^pH unit)	1.2 (0.1–2.9) (*n *= 7)	4.0 (2.3–8.9) (*n *= 13)	<0.001	4.4 (0.5–6.7) (*n *= 10)	5.2 (0.7–10.1) (*n *= 19)	0.171
DS_HAP_	1.4 (0.2–3.6) (*n *= 9)	3.1 (2.5–10.4) (*n *= 16)	0.001	7.3 (0.6–21.4) (*n *= 13)	10.0 (4.5–22.0) (*n *= 19)	0.372
Critical pH	5.4 (5.1–6.2) (*n *= 7)	5.3 (5.0–5.5) (*n *= 16)	0.413	5.5 (5.2–6.2) (*n *= 11)	5.7 (5.2–5.8) (*n *= 19)	0.611

The buffer capacity (β) was calculated from the bicarbonate and phosphate concentrations individually for each saliva sample at its pH value, DS_HAP _denotes the degree of saturation with regard to hydroxyapatite. P-value obtained by a Wilcoxon's rank sum test. * corresponds to mean value below 0.01 ml/min.

**Figure 1:**
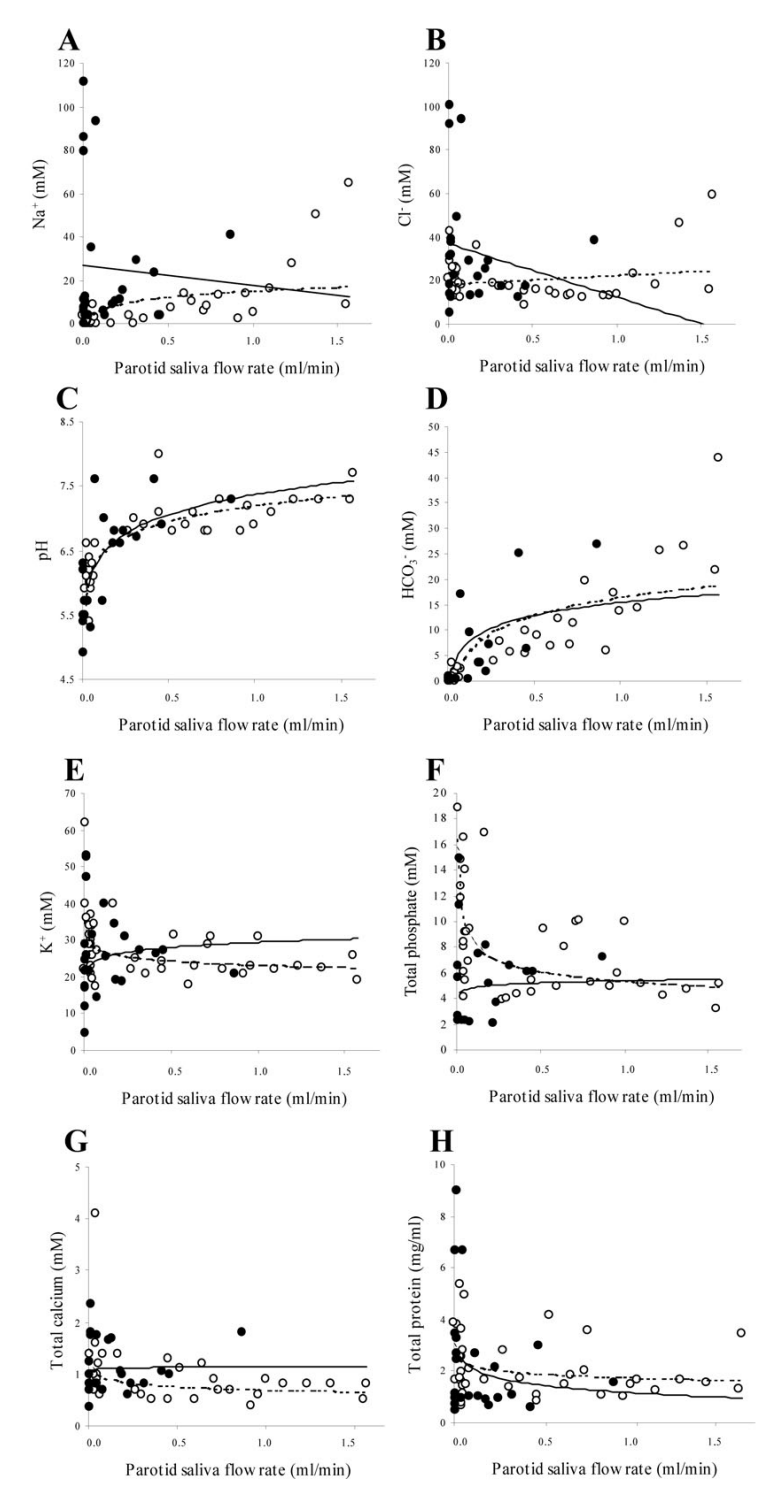
shows the saliva composition as a function of the parotid saliva flow rate (both unstimulated and stimulated) in patients with pSS (bold circles) and healthy age-matched controls (open circles). Lines were fitted by linear (a and b) or non-linear regression (c-h) depending on the best obtainable fit judged from the R-squared values with unbroken lines representing pSS and dotted lines the controls. Figures 1a and b illustrates that the pSS patients can be divided into two subgroups with regard to salt reabsorption. One group of patients follow the reabsorption pattern of the healthy controls, whereas the other group display high sodium and chloride concentrations despite low parotid flow rates indicating differences in the degree of glandular affection by the disease of the patients studied.

In 10, 9, 13 and 7 cases, respectively, volume of UWS, SWS, UPS and SPS samples from pSS patients were insufficient for full sialochemical assessment. In pSS, the concentrations of sodium were significantly higher in both whole and parotid saliva as compared to the healthy controls. Similar results were obtained for chloride concentrations (except for the UPS) (Tables 3 and 4). Regarding the concentrations of the other electrolytes (potassium, total calcium and total phosphate) as well as the organic components (total protein and amylase activity) no statistically significant differences were found between the two groups. In order to test whether the changes in sodium and chloride concentrations were merely related to changes in salivary flow rates, the patients and healthy controls were matched by their UPS and SPS flow rates within the range of 0.01–1.00 ml/min. Figures 1A and 1B illustrate that the pSS patients may be divided into two subgroups. In one group of patients the salt reabsorption pattern was similar to that of the healthy controls, whereas another group displayed high concentrations of parotid sodium and chloride despite low parotid flow rates.

The pH and bicarbonate concentration in parotid saliva were significantly lower in the pSS patients as compared to the healthy controls (Table 4). Subsequently, the pSS patients also had a significantly reduced buffer capacity (β) of UPS and a lower degree of saturation with regard to hydroxyapatite (DS_HAP_) in their UPS than the controls. In pSS, the pH in UPS was in fact only 0.1 pH units from their critical pH. Overall, the salivary β and DS_HAP _were reduced due to low pH, bicarbonate and phosphate concentrations. In pSS, P_CO2 _of SPS was lower than in that of the healthy controls (*P *= 0.02). Nevertheless, at corresponding parotid flow rates, be it stimulated or not, the relations between the flow rates, the saliva pH and the bicarbonate concentrations were similar to those of the healthy controls (Fig. 1C and 1D). Also the concentrations of potassium, total phosphate, total calcium and total protein (Fig. 1E, F, G, and 1H) were similar to those of the controls.

### Determinants of DMFS

As shown in Table 2 the pSS patients had significantly higher DMFS than the healthy controls (p < 0.001) due to higher number of filled (51 ± 23) and missed (37 ± 35) tooth surfaces than the controls (33 ± 19 and 15 ± 35, respectively) (*P *< 0.05). Also the prevalence of dental treatment, i.e. the number of filled surfaces relative to the total number of present tooth surfaces, was significantly higher in the patients (55 ± 27%) than in the controls (25 ± 14%) (*P *< 0.001). However, no significant differences were obtained for the number of decayed surfaces between the patients and controls.

Table 5 shows the explanatory power of the two sets of variables exhibiting the highest explanatory power on DMFS in the pooled patient and control groups, namely complaints of oral dryness and the actual salivary flow rates. The estimate states the theoretical expected increase or decrease in DMFS upon an integer increase of the explaining variables, i.e. one score of subjective oral dryness or one ml of saliva. Furthermore, the subjects' age were added to the analyses due to the well-known effect of this variable upon the number of missing surfaces, which was also found to be significant in this study (*r*_*s *_= 0.63, *P *< 0.001). As shown DMFS increases with increasing age (per year) and increasing complaints of oral dryness, but decreases with increasing salivary flow rates.

**Table 5:** Explanatory power of age as well as subjective and objective salivary variables on DMFS in the patient group and control group all together (*n *= 40).

	Estimate for DMFS	SD for DMFS estimate	Adjusted R-squared	P-value
Subjective model				
Age	1.4	0.4	0.26	<0.001
+ oral dryness questionnaire	11.1	2.3	0.53	<0.001
				
Objective model				
Age	1.4	0.4	0.26	<0.001
+ UWS	-57.4	20.7	0.37	<0.001
+ SWS	-28.9	9.4	0.49	<0.001
+ UPS	-499.2	173.0	0.57	<0.001

Estimate, SD, adjusted R-squared, and P-value obtained by multiple regression analysis. The estimate states the increase or decrease in DMFS upon addition of the explaining variables (age + oral dryness, or + UWS, SWS, and UPS) by an integer (one year, score, or ml). Since complaints of oral dryness and salivary flow demonstrated strong interrelations they could not be included in the same analysis. As shown age in combination with complaints of oral dryness explained 53% of the variance in DMFS, and age combined with salivary flow rates explained 57%.

In the pSS patients, regardless of their age, the complaint of oral dryness, but not the actual salivary flow rate, was associated with dental status (DMFS). The lack of significant correlation between flow rate and DMFS may be ascribed to the narrow window of flow rates in pSS. Scores of oral dryness correlated significantly with the total DMFS (*r*_*s *_= 0.53, *P *< 0.05) and the prevalence of dental treatment (*r*_*s *_= 0.51, *P *< 0.05). In addition, the prevalence of dental treatment increased with the duration of pSS (*r*_*s *_= 0.49, *P *< 0.05), independently of the patient's age.

### Salivary gland focus score, serum autoantibodies, salivary flow and composition

In the pSS group, the focus scores (which ranged from 0 to 4) were significantly inversely correlated to UWS (*r*_*s *_= -0.53, *P *< 0.05), SWS (*r*_*s *_= -0.68, *P *< 0.01), SPS (*r*_*s *_= -0.57, *P *< 0.01), but not to UPS. Focus scores were not correlated to oral dryness, time since pSS diagnosis, and duration of disease symptoms. It is noteworthy, however, that the patient group exhibited large variability with regard to time since diagnosis and age (Table 1). The number of focus scores and presence of serum autoantibodies were positively correlated to salivary concentrations of sodium and chloride in SPS (*r*_*s *_= 0.61, *P *< 0.05 and *r*_*s *_= 0.71, *P *< 0.01). Although highly increased sodium and chloride concentrations were found in UPS as well, the number of observations was too small to generate statistical significance due to the sample volumes that did not allow for sialochemistry (Table 4). Results further revealed that pSS patients with the lowest UWS (i.e., 0–0.05 ml/min) also had the highest focus scores (mean focus score 2.3 *vs*. 0.7, respectively, *P *< 0.05), presence of serum autoantibodies (n = 14), highest concentrations of salivary sodium and chloride, but lowest salivary concentrations of total phosphate compared to patients with UWS ≥ 0.05 ml/min. Pooling data of UPS and SPS for the pSS group allowed us to compare profiles of the patients with high sodium and chloride concentrations to those with low ones. The results of this analysis revealed that pSS with more than 40 mM sodium in their parotid saliva also had significantly higher focus scores as well as presence of serum autoantibodies, than pSS patients below this value (*P *< 0.01). The former also tended to have lower parotid flow rates, more decayed tooth surfaces and to be younger than the other pSS patients.

## Discussion

Oral dryness and reduced salivary flow rates are some of the most predominant and troubling oral sequelae of pSS. This discussion focuses on the hypothesis if impaired salivary flow and/or composition and high caries experience may serve as potential markers of disease severity in pSS.

### Caries experience, oral hygiene, and salivary flow rates

The present study confirms the results of previous cross-sectional studies showing that pSS patients have a significantly higher DMFS compared to healthy controls, despite the fact that these patients often have a good oral hygiene and frequent dental follow-up visits [5,9,10]. When comparing SS patients with patients suffering from other immune diseases and patients with xerostomia of other origins, Boutsi *et al*. [26] found no significant differences in the number of decayed, missed or filled teeth. In accordance with our results, the SS patients were characterised by having lower salivary flow rates, better oral hygiene habits, slightly higher gingival scores, but similar plaque scores, compared to the other groups [26]. Regarding the other periodontal measures, our results support those of previous studies showing that presence of periodontal disease is not substantially increased in pSS [2,26]. The slightly increased gingival scores could be explained by altered inflammatory response probably due to hormonal changes [27]. It has been reported that pSS patients harbour lower numbers of periopathogenic microorganisms than healthy controls [28]. In contrast, and despite a good oral hygiene, pSS patients appear to harbour higher numbers of cariogenic and acidophilic microorganisms such as *Streptococcus mutans *and *Lactobacillus *species than healthy controls [11]. Similar relationships between low salivary flow rates and increased Lactobacillus counts have also been observed in patients with hyposalivation of other origins [29]. Thus, impaired salivary flow and changes in saliva composition as seen in pSS are assumed to favour a more aciduric oral microflora manifested by an increased incidence of caries and fungal infections [28]. At low flow rates, the bicarbonate concentration, pH, and buffer capacity as well as the clearance of microorganisms and dietary sugars in the oral cavity generally decrease [29], thereby promoting an environment dominated by these oral pathogens and prolonged exposure of dietary sugars to the teeth. In pSS patients with severely reduced salivary flow rates the shift in the oral microflora appears to occur despite a good oral hygiene. In addition, the high number of microbial retention sites generated by dental restorations such as fillings, crowns and bridges found in pSS patients may contribute to the shift in oral microflora [28] and complicate the maintenance of sufficient oral hygiene procedures.

In accordance with previous studies we found that the DMFS score is inversely correlated to salivary flow rates and especially the unstimulated whole saliva flow rate [8,10]. However, it has not yet been possible to identify a cut-off value for salivary flow rate that can predict the risk of developing dental caries, as caries is a multifactorial disease. In this study, 90% of the patients had UWS flow rates below 0.10 ml/min, but as this value is merely a part of the classification criteria for pSS, it does not necessarily reflect the point at which caries is likely to develop. It has been suggested that salivary flow rates up to 0.16 ml/min, which include most pSS patients, may result in oral candidiasis [31] and increased development of experimental caries [29]. Meanwhile, the onset of increased caries activity in pSS remains unclear. It has been stated that in patients with SS, loss of teeth due to caries precede the first symptom of xerostomia by on average 9 years [10]. Changes in salivary flow and composition, and subsequently development of caries, appear to precede the symptom of oral dryness by several years. Increased caries activity that cannot be explained by changes in habits related to oral hygiene or diet may therefore represent a useful clinical feature to suspect early pSS in women without complaints of oral dryness, or intake of xerogenic medications. In contrast to the uncertainty pertaining to the onset of pSS and the initial consequences in terms of increased caries lesions, the future perspectives on the patients' dental health seem gloomy without professional preventive intervention. In accordance with the observation that past caries experience is one of the best predictors for future caries [32,33], the pSS patients in this study have a much higher risk of developing future caries and ultimately to loose teeth than the control group. Accordingly, these patients should receive an individual dental care programme in terms of oral hygiene instructions, professional oral hygiene regimens, fluoride treatment, dietary supervision and frequent dental follow-up visits in order to prevent accelerated caries development.

### Self-assessment of oral dryness

In healthy subjects, the sensation of oral dryness usually occurs when whole saliva flow rate is reduced with more than 50% [34]. In this study, all patients had complaints of dry mouth and also substantially decreased salivary flow rates, whereas the healthy controls had no dry mouth complaints and in general, salivary flow rates within the normal range [35].

Nonetheless, in pSS, the scores of oral dryness were only inversely correlated to SWS, which may reflect a larger span of SWS values than of UWS. We have previously found an inverse correlation between scores of oral dryness and UWS [8]. In the present study, the patients had symptoms of oral dryness median 10 years prior to the diagnosis of pSS. Symptoms of oral dryness in combination with the participants' age could explain about half of the variance in DMFS (Table 5). The questionnaire may therefore not only be helpful in assessing the intensity of oral dryness but also in identifying patients with high DMFS.

### Saliva pH and buffer capacity

The ability of human saliva to buffer acids is essential for maintaining pH values in the oral environment above the critical pH for hydroxyapatite (HAP), thereby protecting the teeth against demineralisation. The buffer systems responsible for the human saliva buffer capacity include the bicarbonate, phosphate and protein systems [17,36-38]. In normalcy, where the pH ranges from 6.0 to 7.5, the bicarbonate and phosphate buffer systems are by far the dominant ones having optimal buffering capacity at their pK values of 6.1 and 6.8, respectively [16,19], whereas the proteins have some effect on the buffer capacity at acidic pH values below 5 [16,38].

In this study, the parotid saliva buffer capacity was calculated individually for each pSS patient and healthy control based on the saliva bicarbonate and phosphate concentrations at the respective saliva pH values. This calculation gives an estimate of the buffer capacity of the saliva at the time it is secreted from the parotid gland. In the pSS patients, the buffer capacity of UPS was significantly lower than in the healthy controls, mainly due to the low pH value and bicarbonate concentration in saliva caused by the low flow rates. Moreover, in pSS most of the pH values of UPS were below the relevant pK values of both the bicarbonate and phosphate buffer systems. As compared to healthy controls, the pSS patients will therefore experience far more abundant pH drops in their saliva if exposed to acidic challenges leading to a higher risk of tooth demineralisation. Apart from a low buffer capacity, and thereby impaired ability to maintain a non-acidic saliva pH, the pSS patients also had a significantly lower degree of saturation with respect to HAP in their saliva (Table 4). Thus, in pSS, the mean pH of UPS was only one tenth of a pH unit above their mean critical pH. This implies that even a minor drop in pH will lead to undersaturation of their saliva with respect to HAP and result in either caries lesions or erosive damage to the teeth depending on the origin of the acidic challenge.

### Salivary bicarbonate and phosphate

Despite the differences between pSS patients and healthy controls in sodium and chloride concentrations at comparable low flow rates, stimulated or not, the HCO_3_^- ^concentration and saliva pH did not differ between the two groups (Fig. 1A–D). The transport of bicarbonate in the salivary glands is believed to occur via chloride/bicarbonate exchange mechanisms [39]. The concentration of bicarbonate in saliva is a consequence of the metabolic CO_2 _-turnover in the salivary glands. CO_2 _freely diffuses across the epithelial boundaries, and due to the presence of carbonic anhydrase, the partial pressure for CO_2 _and pH in the glandular compartments governs how much of bicarbonate buffer system is present in form of HCO_3_^- ^in the saliva. The duct epithelium has dual functions with respect to bicarbonate transport, since it can both reabsorb (at low secretion rates) and secrete (at high secretion rates due to increased metabolic turnover of the gland).

This study demonstrated a tendency towards lower total phosphate concentrations in pSS patients than in healthy controls. Previous studies have found significantly reduced phosphate concentrations in stimulated parotid and SM/SL saliva of patients with SS compared to healthy controls and patients with conditions resembling SS [40-42]. It should be stressed that the mechanism behind phosphate transport in human salivary gland tissues has not yet been fully characterised. It probably includes an acinar secretion and/or a ductal reabsorption via sodium-phosphate co-transport mechanism as that observed in the renal proximal tubules [43].

### Salivary sodium and chloride

Our sialochemical results are interpreted within the frame of the classical two-stage model of saliva formation [44]. Under normal physiological conditions, and in response to nervous stimuli, the acinar cells produce primary saliva, which has an ionic composition resembling that of plasma. As the primary saliva passes through the duct system it becomes modified by reabsorption of sodium and chloride (but without water due to the low water permeability) whereby the final saliva secreted into the oral cavity becomes hypotonic with sodium and chloride concentrations much below that of the original primary saliva. The composition of the final saliva secreted into the oral cavity strongly depends on the secretion rate in such a way that at low flow rates the saliva contains low sodium and chloride concentrations and as the flow rates increase the concentrations of sodium and chloride will rise. This normal physiological relation between parotid flow rate and sodium and chloride concentrations was seen in the healthy controls and in some of the pSS patients (0.01–1.00 ml/min) as well (Fig. 1A and 1B). However, for other pSS patients with flow rates within the same frame, another picture emerged, since their sodium and chloride concentrations were remarkably higher than normally whether stimulated or not. Thus, on a group basis the pSS patients have significantly higher concentrations of sodium and chloride than the healthy controls (Table 4). This finding is in accordance with several previous studies on whole saliva, parotid and submandibular/sublingual saliva (SM/SL) [5,40-42,45,46]. The concentrations of sodium and chloride have also been shown to be higher in SM/SL of patients with pSS and secondary SS compared to patients with clinical conditions resembling SS, i.e., sialoadenosis, sodium retention dysfunction syndrome and medication-induced xerostomia [42]. Overall, these compositional changes appear to be unique for some pSS patients. It has been stated that SM/SL glands are affected earlier by SS than the parotid glands due to an average reduction of stimulated SM/SL flow rate preceding that of the stimulated parotid flow rate [40,42]. On the other hand, cut-off values for sodium, chloride and phosphate in SPS and stimulated SM/SL being predictive for SS demonstrated almost similar specificity (69 and 71%, respectively) and sensitivity (81%) [32].

### Other salivary constituents

Despite the low salivary flow rates seen in the pSS, the acinar transport mechanisms involved in the formation of primary saliva seem to be unaffected by the glandular lymphocytic infiltration. Accordingly, concentrations of potassium, total calcium, total protein and amylase activity in whole and parotid saliva did not differ from those of the healthy controls, which is in agreement with previous reports [40,45]. Furthermore, it has been shown that the output of statherin and acidic proline-rich proteins, which reflect the secretion of selected parotid proteins, did not differ between pSS patients and healthy controls [5]. Normal concentrations of total calcium, total protein and levels of amylase activity indicate that the remaining functional acinar cells are capable of synthesis and secretion of primary saliva with normal composition despite the marked lymphocytic infiltration and structural changes.

### Impaired in ductal salt reabsorption reflects disease severity in pSS

The changes in salivary composition indicate that the duct epithelium, and mainly the striated duct epithelium, cannot effectively reabsorb the high concentrations of sodium and chloride of the primary saliva in some of the pSS patients, despite low salivary flow rates. The pSS patients who exhibited high concentrations of sodium and chloride were also characterised by having the lowest flow rates, the highest focus score (FS) and highest concentrations of serum autoantibodies (AB) in addition to a tendency of being younger than the pSS patients with normal salivary concentrations of sodium and chloride. Overall, the subgroup of pSS patients with the highest concentrations of salivary sodium and chloride and lowest total phosphate concentrations appeared to be more severely affected by pSS having more exocrine and non-exocrine disease manifestations than those without these salivary changes supporting previous observations [5]. It should be stressed that there was no relationship between reduced salivary flow and compositional changes and intake of medications. The question is whether the subgroup of patients with normal salivary concentrations of sodium and chloride and some preserved salivary gland function has a late onset of pSS or a "milder" glandular response to autoimmunity compared to the subgroup with high salivary sodium and chloride concentrations or salivary secretion close to or equal to 0 ml/min. Another relevant question is whether the latter subgroup of patients also has a greater risk of developing malignant lymphoma. The results of our study therefore need to be tested in a large prospective cohort study and compared with group of patients with non-immunological destruction of their salivary glands in order to validate the use of specific salivary changes and dental caries as markers of salivary gland dysfunctional severity/disease severity in pSS. At present there is no international expert consensus regarding measures for assessment of disease activity, severity, damage or outcome in pSS that can be used in the evaluation of clinical trials of new therapies and longitudinal observational studies. Recent reports, however, indicate that the development and evaluation of such measures has begun [47,48].

In pSS, the salivary gland histopathology is characterised by periductal lymphocytic infiltration and acinar destruction. The duct epithelium, however, appears relatively unaffected by the lymphocytic infiltration, which contrasts the observed salivary changes in pSS. On the other hand, the discrepancy may be explained by morphological differences between the labial salivary glands, which predominantly consist of mucous acini and have a very short duct system, and the parotid glands, which comprise serous acini and have long duct system. Nevertheless, it has been stated that the histopathological changes of the labial salivary glands mimic those of the parotid glands [49,50]. It has been suggested that Bcl-2 positive basal cells of striated/excretory ducts possess an extensive capacity for pluridirectional morphogenetic differentiation [51]. On this basis, it could be speculated that the duct cells in some pSS patients possess antigenic properties, which initiate an autoimmune response that could be associated with morphogenesis and cell differentiation of the salivary gland tissues. It has not yet been possible to identify a specific anti-salivary duct antibody in pSS that is capable of inhibiting cell differentiation. Our finding of more pronounced acinar and ductal tissue functional impairment in pSS patients with both FS and AB, than in patients with FS or AB, supports the idea that circulating autoantibodies or inflammatory mediators produced locally by the inflammatory cells, interfere with the neural release of neurotransmitter substances or interact with the binding of neurotransmitters to receptors on the cell surface, thereby impairing the acinar secretion and/or the ductal reabsorptive modification of saliva [2]. Along this line, an *in vitro *study on isolated human acini and duct segments from pSS patients have shown that these cells possess functional receptor systems and normal response in changes in the intracellular free calcium concentration upon maximal secretagogue stimulation [52]. The fact that the ductal uptake of sodium *via *amiloride-sensitive epithelial sodium channel (ENaC) is regulated by circulating adrenal mineralocorticoids, e.g. aldosterone [53] could also indicate that some pSS patients have low levels of aldosterone. However, this still needs to be clarified.

## Conclusion

The results of this study indicate that specific sialometric and sialochemical glandular changes, particularly changes in sodium and chloride concentrations, may serve as oral markers of disease severity in pSS. The question arises whether pSS represents a continuum of patients with different stages of disease/affection of salivary glands or different diseases affecting the salivary gland tissues. There is great need of disease assessment and outcome markers in pSS. The hypotheses generated from our results on changes in salivary flow and composition as well as high caries experience as potential markers of the extent of autoimmune-mediated salivary gland dysfunction in pSS therefore need to be tested in a large prospective cohort study including patients with early to long-standing disease. In addition, the mechanisms underlying the impaired ductal salt reabsorption observed in the pSS patients with presence of both labial salivary gland focus score and serum-autoantibodies need to be further elucidated in future pathophysiological studies.

## Abbreviations

SS = Sjögren's syndrome; pSS = primary Sjögren's syndrome; FS = focal lymphocytic infiltration in the labial salivary glands; UWS = unstimulated whole saliva flow rate; SWS = stimulated whole saliva flow rate; UPS = unstimulated parotid saliva flow rate; SPS = stimulated parotid saliva flow rate; AB = presence of serum autoantibodies to Ro (SSA) and/or La (SSB) antigens; DMFS = decayed, missing and filled tooth surfaces; PI and GI = plaque and gingival indices; PPD = periodontal probing pocket depth; HAP = hydroxyapatite.

## Competing interests

The author(s) declare that they have no competing interests.

## Authors' contributions

All three authors participated in the design of the study and the statistical analysis. AMLP attended coordination of the study and conducted the whole saliva collections, interviews, oral clinical examinations and labial salivary gland biopsies. AB performed the parotid saliva collections and sialochemical analyses. All authors read and approved the final manuscript.

## Pre-publication history

The pre-publication history for this paper can be accessed here:


